# Modulation of CP2 Family Transcriptional Activity by CRTR-1 and Sumoylation

**DOI:** 10.1371/journal.pone.0011702

**Published:** 2010-07-22

**Authors:** Sarah To, Stephen J. Rodda, Peter D. Rathjen, Rebecca A. Keough

**Affiliations:** 1 School of Molecular and Biomedical Science, University of Adelaide, Adelaide, South Australia, Australia; 2 ARC Special Research Centre for the Molecular Genetics of Development, University of Adelaide, Adelaide, South Australia, Australia; 3 Department of Zoology, University of Melbourne, Melbourne, Victoria, Australia; Cincinnati Children's Hospital Medical Center, United States of America

## Abstract

CRTR-1 is a member of the CP2 family of transcription factors. Unlike other members of the family which are widely expressed, CRTR-1 expression shows specific spatio-temporal regulation. Gene targeting demonstrates that CRTR-1 plays a central role in the maturation and function of the salivary glands and the kidney. CRTR-1 has also recently been identified as a component of the complex transcriptional network that maintains pluripotency in embryonic stem (ES) cells. CRTR-1 was previously shown to be a repressor of transcription. We examine the activity of CRTR-1 in ES and other cells and show that CRTR-1 is generally an activator of transcription and that it modulates the activity of other family members, CP2, NF2d9 and altNF2d9, in a cell specific manner. We also demonstrate that CRTR-1 activity is regulated by sumoylation at a single major site, residue K30. These findings imply that functional redundancy with other family members may mask important roles for CRTR-1 in other tissues, including the blastocyst stage embryo and embryonic stem cells.

## Introduction

The CP2 transcription factor family forms one branch of the grainyhead-related protein family [1]. CP2 (also known as LSF and LBP-1c in humans), its splice variant CP2d (also referred to as LSF1d or LBP-1d in humans), NF2d9 (referred to as LBP-1a in humans), its splice variant altNF2d9 (LBP-1b in humans) and CRTR-1 (also known as Tcfcp2l1 and TFCP2L1 or LBP-9 in humans) comprise this branch. CP2 and NF2d9 are widely, if not ubiquitously, expressed. Both NF2d9 and its splice variant, altNF2d9, generally act as transcriptional activators [2], and CP2 can activate or repress transcription [1]. In contrast, CRTR-1 was reported to be a specific repressor of transcription [3], and its expression is regulated both developmentally and tissue-specifically. Major sites of CRTR-1 expression include the early mammalian blastocyst, embryonic stem (ES) cells and developing and adult exocrine glands, particularly kidneys and salivary glands [3], [4], [5], [6]. Gene targeting of CRTR-1 in mice results in postnatal lethality of up to 70% of mice, presumably due to renal failure caused by defective duct maturation [4].

Mammalian CP2 family proteins are encoded by three separate genes and all share high levels of amino acid sequence similarity (83% or greater similarity between mouse CP2, NF2d9 and CRTR-1). As such, it is predicted that members of the family will recognise the same DNA motif (CNRG-N_6_-CNRG) [1] and bind DNA as tetramers [7], [8], forming either homomeric complexes or heteromeric complexes with other family members, as has been demonstrated for mouse CP2 and the human LBP-1a, b and c proteins [2], [7], [8], [9].

Several recent studies have implicated CRTR-1 (Tcfcp2l1) in the complex transcription factor network responsible for the maintenance of pluripotency in mouse ES cells. CRTR-1 has been shown to bind to the regulatory regions of the *Oct4* (*Pou5f1*), *Nanog*, *Sox2* and *Klf4* genes [10], which are core components of this network. The *CRTR-1* gene itself appears to be regulated by pluripotency factors, with demonstrated binding of Oct4, Nanog and Jmjd1a, a histone demethylase required for pluripotency, to upstream regions [11], [12]. Despite a putative role in the expression of genes required for pluripotency, the activity of CRTR-1 in ES cells has not been tested to date.

We examine the activity of CRTR-1 in ES cells and also in the kidney cell lines, COS-1 and HEK293T. We demonstrate that CRTR-1 binds DNA and activates transcription through a CP2-response element and show that it interacts with, and modulates the activity of, other CP2 family proteins resulting in enhancement or suppression of activity depending on the CP2 family member and cell type. Moreover, we show that CRTR-1 can be sumoylated and that this modification regulates its activity. These findings demonstrate the potential for functional redundancy between CRTR-1 and other family members and suggest that activity should be considered in terms of the CP2 family profile in a given cell, rather than that of an individual family member.

## Results

### CRTR-1 can act as a transcriptional activator

**Figure 1:**
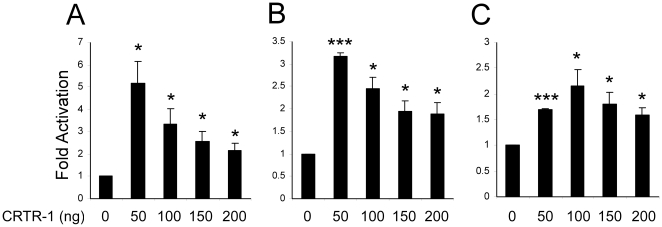
CRTR-1 activates transcription in a cell type specific manner. ES (A), HEK293T (B), or COS-1 (C) cells were co-transfected with pTK-4xWT-CP2-LUC and pEF-CRTR-1expression plasmid (CRTR-1) as indicated. Relative luciferase activity was determined by normalisation to renilla activity. The data are presented relative to the activity of the reporter vector alone and are the mean ± SEM of three independent experiments, each of which was conducted in triplicate. Statistical significance was determined using a two-tailed unpaired t-test comparing the activities of different amounts of CRTR-1 with vector alone. * denotes statistical significance with P<0.05, and *** denotes statistical significance with P<0.0001. Also see supplementary data Figure S1.

Many transcription factors have the ability to both activate and repress transcription, as is seen for CP2 [1]. However, CRTR-1 and LBP-9 have been characterised as specific transcriptional repressors [3], [13], [14]. To investigate the activity of CRTR-1 in ES cells, a CP2-responsive luciferase reporter construct (pTK-4xWT-CP2-LUC) was co-transfected with increasing amounts of a CRTR-1 expression plasmid (pEF-CRTR-1) into ES cells (Figure 1A and Figure S1A). CRTR-1 was able to activate transcription at all concentrations of CRTR-1 plasmid. Highest activation levels, up to 5 fold, were obtained with lower amounts of CRTR-1 plasmid. To determine if activation was cell type specific, CRTR-1 activity was also examined in HEK293T and COS-1 cells. Up to 3 fold activation was observed in HEK293T cells, with maximal activity when lower amounts of CRTR-1 plasmid were used (Figure 1B). In COS-1 cells, CRTR-1 activity was lower (Figure 1C). Although statistically reproducible, more than 2 fold activation was rarely observed, suggesting cell type-specific activity. (CRTR-1 expression levels in the three cell types are shown in Figure S1B). In all cell types, CRTR-1 activity is mediated through the CP2 binding elements in the reporter construct, as mutation of these sites abrogates activity (data not shown).

### Mapping of activation/repression regions of CRTR-1

**Figure 2:**
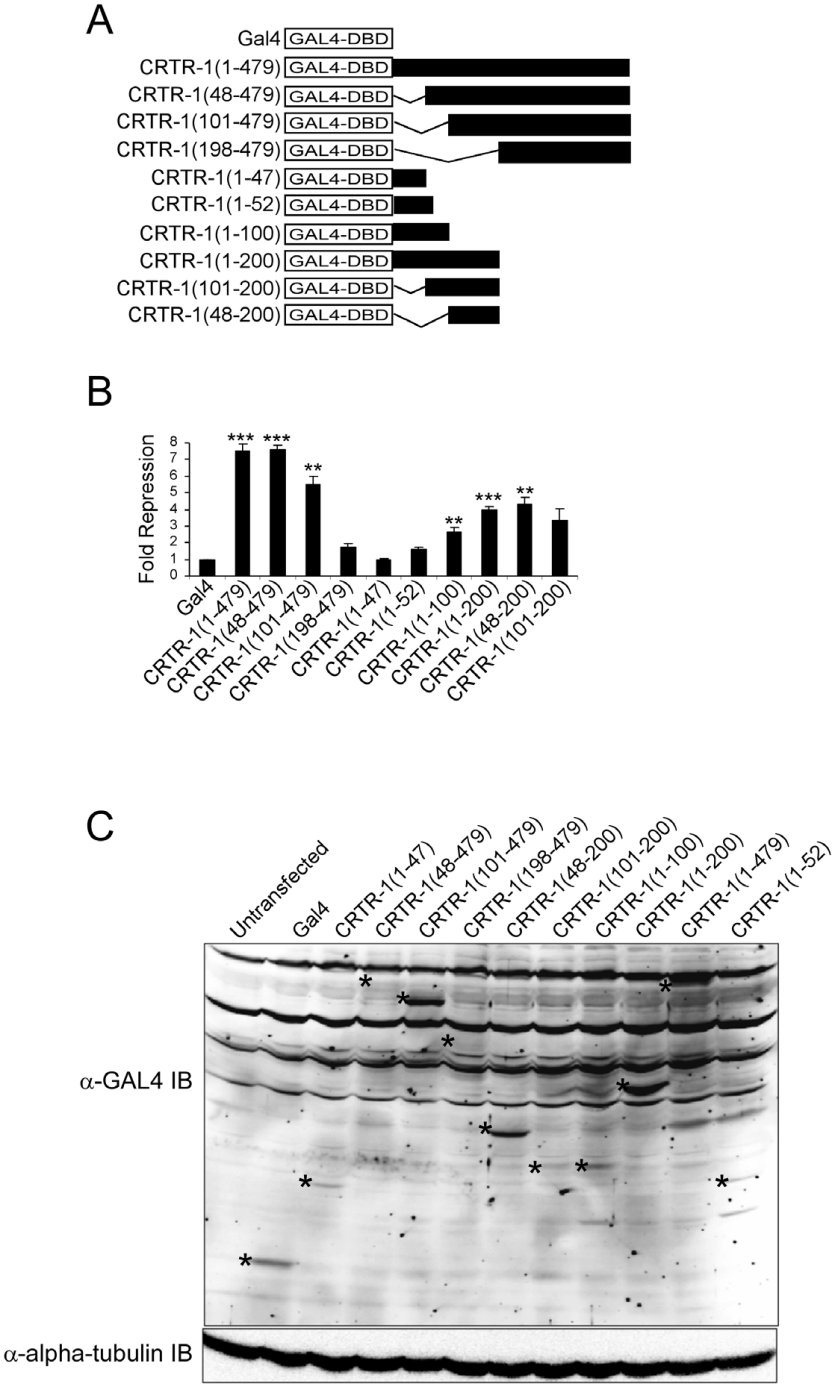
Mapping of CRTR-1 transactivation and repression domains. *A.* Schematic diagram of the CRTR-1 truncations fused to the GAL4 DNA-binding domain. Numbers in brackets represent the first and last amino acid of the CRTR-1 protein included in the protein. *B.* Luciferase reporter assays of GAL4-CRTR-1 deletion constructs. HEK293T cells were co-transfected with 200 ng pTK-MH100x4-LUC and 200 ng of expression plasmid for the GAL4-CRTR-1 deletion mutants together with 5 ng of pRL-SV40 for normalizing transfection efficiency. The data are presented relative to the activity of the reporter construct alone and are the mean ± SEM of three independent experiments, each of which was conducted in triplicate. Statistical significance was determined using a two-tailed unpaired t-test comparing the activities of each GAL4-CRTR-1 deletion mutant with GAL4 vector alone. ** denotes statistical significance with P<0.01; *** denotes P<0.0001. *C.* Expression level of GAL4-CRTR-1 deletion mutants. Whole cell lysates from HEK293T cells transfected with 2 µg of expression plasmid encoding GAL4-CRTR-1 deletion mutants were immunoblotted with an anti-GAL4 antibody and detected by ECF (upper panel). The membrane was re-probed with rat anti-alpha-tubulin antibody and detected by enhanced chemiluminescence (lower panel). The predicted sizes of the GAL4-CRTR-1 fusion proteins are as follows: GAL4-CRTR-1(1–47), 21 kD; GAL4-CRTR-1(48–479), 64 kD; GAL4-CRTR-1(101–479), 58 kD; GAL4-CRTR-1(198–479), 47 kD; GAL4-CRTR-1(48–200), 33 kD; GAL4-CRTR-1(101–200), 27 kD; GAL4-CRTR-1(1–100), 27 kD; GAL4-CRTR-1(1–200), 38 kD; GAL4-CRTR-1(1–479), 69 kD; and GAL4-CRTR-1(1–52), 22 kD. Specific bands corresponding to GAL4-CRTR-1 deletion mutants are marked with an asterisk. Mass of molecular weight markers (kD) are shown.

The repression domain of CRTR-1 had been mapped previously to the extreme N-terminus (amino acids 1–52) [3]. In contrast, the region that mediates repression in the human homologue, LBP-9, has been shown to lie between residues 100 and 200 [13]. However, no activation was seen in these experiments. In an attempt to identify residues responsible for transcriptional activation in CRTR-1 and clarify the region responsible for repression, a more comprehensive series of CRTR-1 deletions fused to the GAL4 DNA binding domain were tested for their ability to repress or activate a GAL4-responsive luciferase reporter construct pTK-MH100x4-LUC (Figure 2). Western blotting (Figure 2C) shows that all proteins were expressed, although levels varied considerably between proteins. Maximal repression (approximately 7 fold) was observed with full length CRTR-1 and CRTR-1(48-479) (Figure 2B), although CRTR-1(48–479) is expressed at lower levels (Figure 2C). The expression levels of CRTR-1(198–479) and CRTR-1(48–479) are similar, but only CRTR-1(48–479) represses. Together, these data suggest that the region responsible encompasses residues 48–198. This is supported by CRTR-1(1–200) which is expressed at levels similar to that of CRTR-1(101–479) and represses well. Repression by CRTR-1(48–200) is comparable to that of CRTR-1(1–200), although it is expressed at lower levels. Despite being a well characterised assay for mapping activation and repression motifs, including those in other CP2 family members [2], [13], we were unable to detect any region of CRTR-1 with transactivation properties using this method. (This is unlikely to be due to the differences in protein levels as there was no consistent correlation between activity and expression level in these experiments).

### CRTR-1 interacts with CP2 family members and forms heteromeric DNA binding complexes

**Figure 3:**
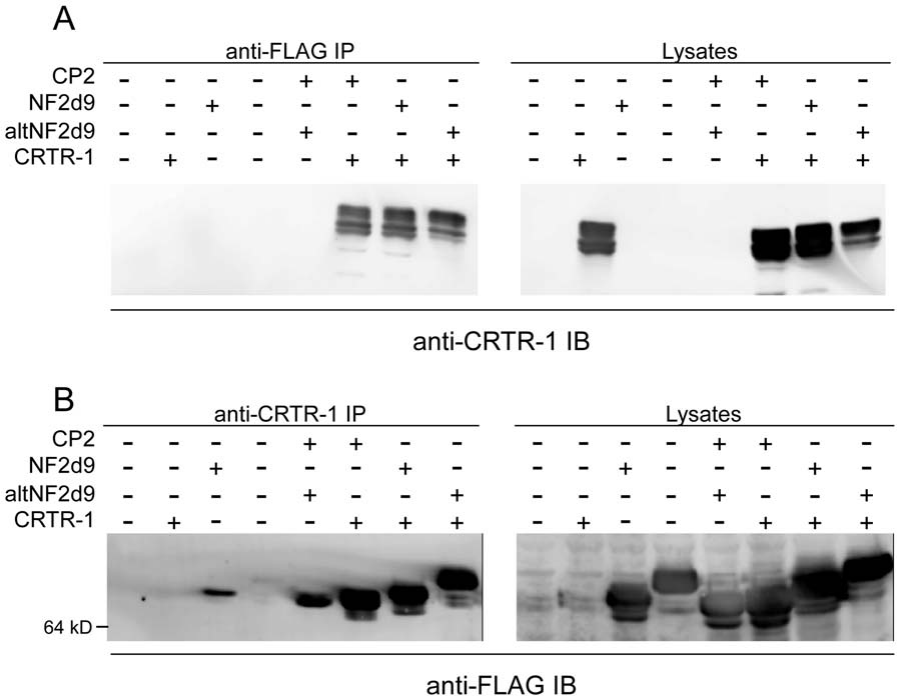
CRTR-1 co-immunoprecipitates with other CP2 family members. HEK293T cells were co-transfected with pEF-IRES-puro6 expression plasmids encoding CRTR-1 and FLAG-CP2, FLAG-NF2d9 or FLAG-altNF2d9, as indicated. Whole cell lysates were immunoprecipitated (IP) with (A) anti-FLAG (M2) antibody or (B) anti-CRTR-1 antibody and immunoblotted (IB) with anti-CRTR-1 or anti-FLAG antibodies respectively. Western blot analysis of input lysates is shown.

CP2 family proteins interact with each other, forming homomers and heteromers [9], with the functional DNA-binding unit thought to be tetrameric [7], [8]. To determine whether CRTR-1 could interact with other CP2 family members, co-immunoprecipitations were performed (Figure 3) using FLAG-tagged CP2, NF2d9 and altNF2d9 co-expressed with CRTR-1 in HEK293T cells. CRTR-1 co-precipitated with all CP2 family proteins (Figure 3A) and this interaction was confirmed with the reciprocal co-immunoprecipitation (Figure 3B). Some background precipitation of FLAG-CP2 and FLAG-NF2d9 was evident (see left-hand panel of Figure 3B). Control experiments performed using pre-immune serum or no antibody (data not shown) demonstrated that this was due to non-specific binding of the FLAG-tagged protein to the agarose beads. However, the greatly enriched pull-down of FLAG-CP2 in the presence of ectopically expressed CRTR-1 indicates that the interaction between CRTR-1 and the other CP2 family members is genuine.

**Figure 4:**
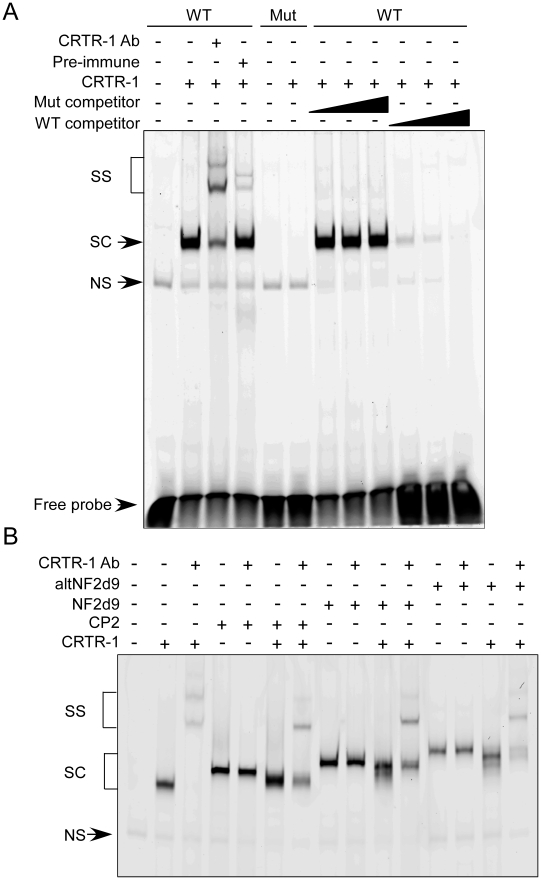
CRTR-1 binds the CP2 response element and forms DNA-binding complexes with other CP2 family members. *A.* Nuclear extracts prepared from untransfected or pEF-CRTR-1 transfected HEK293T cells were incubated with FAM-labelled DNA probe containing a wild type (WT) or mutated (Mut) CP2 response element. Binding reactions were performed in the presence or absence of anti-CRTR-1 antibody or pre-immune serum (pre-immune) as indicated. Competitions were performed in the presence of 50-, 100-, or 200 fold excess unlabelled competitor oligo containing the wild type or mutated CP2 response element. *B*. Nuclear extracts prepared from HEK293T over-expressing CRTR-1 and the indicated CP2 family member were incubated with FAM-labelled DNA probe containing a wild type α-globin CP2 response element in the presence or absence of anti-CRTR-1 antibody. Probe was in excess in all samples. Protein-DNA complexes were resolved by electrophoresis on a 4% native polyacrylamide gel. SC, specific complex; SS, super-shifted complex; NS, non-specific complex.

EMSA experiments demonstrated that CRTR-1-containing complexes could specifically bind DNA containing a CP2-response element (Figure 4A), as no competition for binding was observed using an oligonucleotide containing a mutated CP2-response element. The presence of CRTR-1 protein in the specific complex was shown by super-shift using the CRTR-1 antibody. (Note: the feint super-shifted bands seen with pre-immune serum do not migrate at the same mobility as the super-shifted bands observed with the CRTR-1 anti-serum). EMSA was also used to show that heteromeric DNA-binding complexes are formed with CRTR-1 and CP2, NF2d9 or altNF2d9 as demonstrated by the intermediate mobility between that of CRTR-1 alone and the slower migrating CP2, NF2d9 or altNF2d9 complexes (Figure 4B).

### CRTR-1 modulates the activity of other CP2 family members

**Figure 5:**
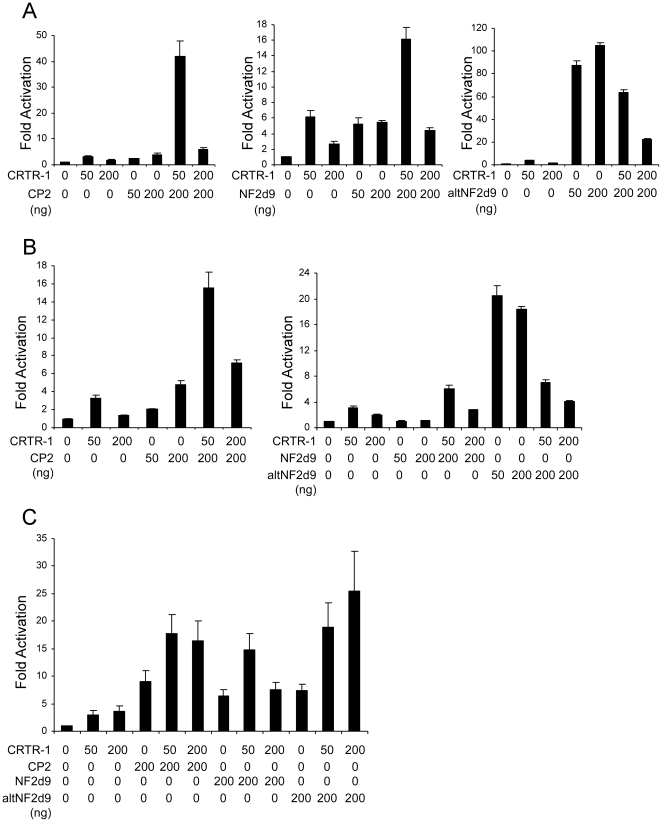
Modulation of CP2, NF2d9 and altNF2d9 activity by CRTR-1. ES cells (A), HEK293T cells (B) and COS-1 cells (C) were co-transfected with pTK-4xWT-CP2-LUC and the indicated amounts of expression plasmids encoding CRTR-1 and FLAG-tagged CP2, NF2d9 or altNF2d9. Relative luciferase activity was determined by normalisation to renilla activity. Data are presented relative to the activity of the reporter construct alone and are the mean ± SEM of three independent experiments, each of which was conducted in triplicate. Also see supplementary Figure S2 for western blots showing protein levels.

To determine the effect of CRTR-1 on the transactivating ability of CP2, NF2d9 and altNF2d9, reporter assays were performed in ES, HEK293T and COS-1 cells (Figure 5; also see Figure S2). Multiple cell lines were chosen to examine possible cell specific effects. Co-transfection of CRTR-1 and CP2 resulted in enhanced transactivation, with increases of up to 12 fold over that of CP2 or CRTR-1 alone in ES cells (Figure 5A) and approximately 3–7 fold in HEK293T cells (Figure 5B). This effect was lost when higher amounts of CRTR-1 plasmid were used (such as 150 ng and 200 ng; data not shown). More modest increases in transactivation were observed when CRTR-1 and NF2d9 were co-transfected (up to 3 fold and 5 fold in ES and HEK293T cells respectively), and only when lower amounts of CRTR-1 plasmid were used. In contrast, co-transfection of CRTR-1 and altNF2d9 resulted in a reduction in luciferase levels compared with altNF2d9 alone, indicating that CRTR-1 is able to suppress altNF2d9-mediated activation (Figure 5A, B). In COS-1 cells, co-expression of CRTR-1 with CP2 or NF2d9 resulted in a modest enhancement of activation, generally at lower amounts of CRTR-1 (up to 2 fold over CP2 or NF2d9 alone; Figure 5C). However, co-expression with altNF2d9 resulted in up to a 3.5 fold increase in transactivation over that of altNF2d9 alone, not the suppression of activity that was observed in ES and HEK293T cells, demonstrating cell type-specific activity. These data show that CRTR-1 is able to modulate the transcriptional activity mediated by other CP2 family proteins, acting to enhance or suppress transactivation depending on the family member and cell type.

### CRTR-1 is sumoylated at lysine 30

**Figure 6:**
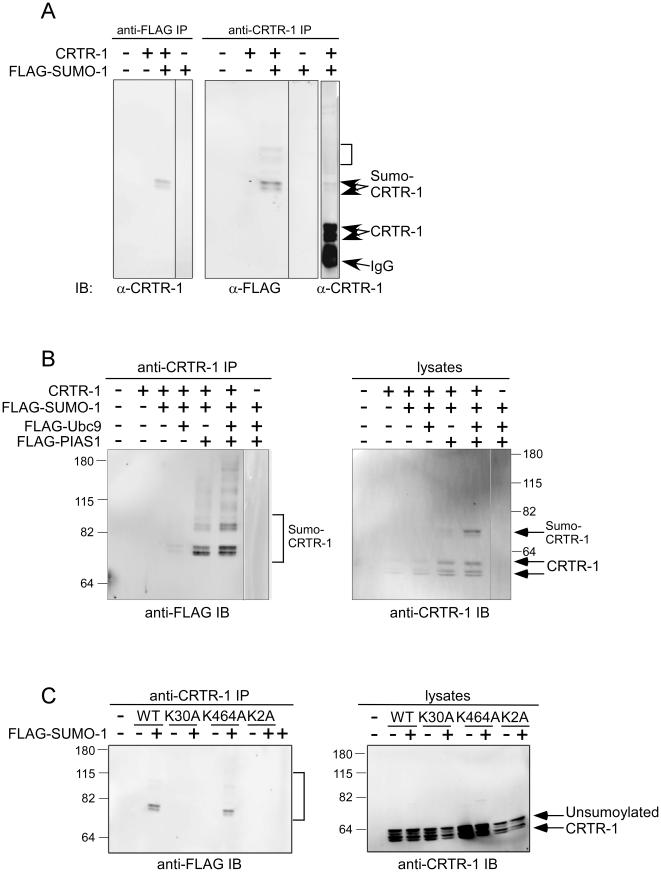
CRTR-1 is sumolyated at K30 and sumoylation is enhanced by PIAS1 or Ubc9. *A.* COS-1 cells were co-transfected with pEF-CRTR-1and pEF-FLAG-SUMO-1 expression plasmids as indicated. Whole cell lysates were immunoprecipitated (IP) with anti-FLAG or anti-CRTR-1 antibody and analysed by immunoblotting (IB) as indicated to detect sumoylated proteins. Bracket identifies higher molecular weight bands as possible multiple or polysumoylated CRTR-1. *B.* COS-1 cells were co-transfected with pEF-CRTR-1, pEF-FLAG-SUMO-1, pEF-FLAG-Ubc9, and pEF-FLAG-PIAS1 expression plasmids as indicated. Whole cell lysates were immunoprecipitated with anti-FLAG or anti-CRTR-1 antibody and analysed by immunoblotting with anti-CRTR-1 or anti-FLAG antibody respectively to detect sumoylated proteins (bracket). Immunoblotting of input lysates with anti-CRTR-1 antibody detected both sumoylated and unsumoylated CRTR-1. *C.* COS-1 cells were co-transfected with pEF-CRTR-1, pEF-K30A, pEF-K464A or pEF-2KA together with pEF-FLAG-SUMO-1 expression plasmids. Whole cell lysates were immunoprecipitated with anti-CRTR-1 antibody and analysed by immunoblotting with anti-FLAG antibody respectively to detect sumoylated proteins (bracket). Immunoblotting of input cell lysates with anti-CRTR-1 antibody detected unsumoylated CRTR-1. Molecular weight markers are shown (kD). Also see Figure S3A which is a re-probing of the blot in (C) with anti-CRTR-1 antibody.

Cell specific behaviour of CRTR-1 is demonstrated by its lower activity in COS-1 cells compared with ES and HEK293T cells and by its ability to enhance, rather than suppress, altNF2d9 activity in this cell type. While this may be due to a number of possible factors, it is well documented that sumoylation of transcription factors can affect their activity and it has also been reported that erythroid-specific activity of CP2 proteins is mediated, in part, by PIAS1, a sumo E3 ligase [2]. We, therefore, investigated the possible role of sumoylation of CRTR-1 on its activity. To determine whether CRTR-1 can be sumoylated, FLAG-SUMO-1 and CRTR-1 expression plasmids were co-transfected into COS-1 cells and whole cell lysates were immunoprecipitated with either FLAG or CRTR-1 antibodies (Figure 6A). Unsumoylated CRTR-1 runs as 2 doublets of approximately 54 and 60 kD. Immunoprecipitated proteins of approximately 75–80 kD are detected with both the CRTR-1 and FLAG antibodies, corresponding to sumoylated forms of CRTR-1. Co-transfection with Ubc9, an E2 sumo conjugating enzyme, or PIAS1 expression plasmids enhanced the level of CRTR-1 sumoylation observed (Figure 6B).

Analysis of the CRTR-1 amino acid sequence identified 2 potential sites for sumoylation, FK^30^QE and LK^464^AE, conforming to the consensus sequence, ψKXE (where ψ is a hydrophobic amino acid and X is any amino acid) [15]. Mutation of the lysine residue in each motif to alanine demonstrated that lysine 30 (K30) is the major site of sumoylation in CRTR-1, with no detectable sumoylation of CRTR-1 when this residue is mutated (Figure 6C).

### Sumoylation alters CRTR-1 activity

**Figure 7:**
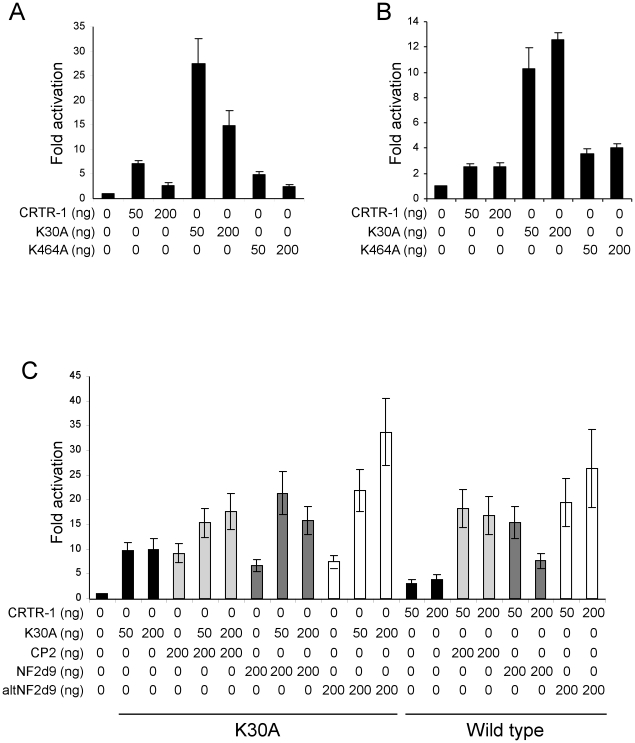
The effect of K30 mutation on CRTR-1 activity. ES cells (A) and COS-1 cells (B) were co-transfected with the indicated amounts of pEF-CRTR-1, pEF-K30A, pEF-K464A or pEF-2KA expression plasmids together with pTK-4xWT-CP2-LUC reporter construct. Relative luciferase activity was determined by normalisation to renilla activity. The data presented are representative experiments of three independent experiments, each of which was conducted in triplicate. Error bars represent the SEM of the triplicate samples. *C*. The effect of the K30A mutant on the activity of CP2, NF2d9 and altNF2d9 was examined in COS-1cells. Cells were co-transfected with the indicated amounts of pEF-K30A, pEF-K464A or pEF-CRTR-1, expression plasmids for CP2, NF2d9 or altNF2d9 and pTK-4xWT-CP2-LUC reporter construct. Firefly luciferase activity was normalised to renilla activity. The data are presented relative to the activity of the reporter vector alone and are the combined data from three independent experiments (±SEM), each performed in triplicate. Shading has been used to aid comparison between the effects of wild type and K30A CRTR-1.

The CRTR-1 sumoylation mutants were tested for their transactivation/suppression ability in reporter assays in ES and COS-1 cells. All sumoylation mutants were expressed at levels similar to that of wild-type CRTR-1 (see Figure S3). The activity of the K30A mutant is up to 4 fold greater than that of wild type CRTR-1in ES cells (Figure 7A), and similar activity is also observed with the K2A mutant (data not shown). The K464A mutant has comparable activity to wild type, consistent with K30 being the critical residue for sumoylation. Interestingly, unlike wild type CRTR-1 which has limited activity in COS-1 cells, the K30A mutant is able to activate the reporter construct to levels approximately 9 fold greater than basal levels (Figure 7B), suggesting that sumoylation of CRTR-1 in COS-1 cells abrogates its ability to activate transcription. The effect of the K30A mutation on the ability of CRTR-1 to modulate the activity of other family members was tested in COS-1 cells (Figure 7C). Interestingly, despite greater activity of K30A CRTR-1 alone, overall activity when K30A CRTR-1 was co-transfected with CP2, NF2d9 or altNF2d9 was not statistically significantly different from that seen on co-transfection of CP2 family members with wild type CRTR-1.

## Discussion

The results presented here demonstrate that CRTR-1 is able to act as a transcriptional activator or suppressor, depending on the cell type and the complement of other CP2 family members present. It is also likely that individual promoter context would play a role in determining CRTR-1 activity, but this has not been addressed here. We show for the first time that CRTR-1 is able to activate transcription. This supports the indirect evidence that LBP-9, the human homologue, may also both activate and repress [13]. Using a GAL4-based assay we were unable to identify a region of CRTR-1 responsible for mediating activation. A similar result was seen for LBP-9 [13]. This could be due to a number of factors including the possibility that fusion to the GAL4 DNA-binding domain obscured identification of an activation domain at the amino-terminus, or that CRTR-1 lacks a classical activation domain and acts by altering the DNA-binding affinity or conformation of partner proteins within the DNA-binding heteromer to elicit activation or repression. The fact that activation domains have been identified at the amino termini of CP2, NF2d9 and altNF2d9 using similar methods [2] adds weight to the possibility that CRTR-1 lacks an activation domain. Such a model would imply that activation by CRTR-1 alone is mediated by incorporation of endogenous CP2, NF2d9 or altNF2d9 into the tetrameric complex, or by recruitment of activators to the homomeric CRTR-1 complex. Further studies into the mechanisms of activation and repression by CRTR-1 are required to resolve this. Similarly, the observation that maximal activation correlates with lower levels of ectopic CRTR-1 expression and that activation decreases with increasing CRTR-1 levels, suggests that the stoichiometry of the CRTR-1-containing complexes may be an important mechanism for modulating CP2-family activity.

The GAL4-based studies mapped the region of CRTR-1 able to mediate repression to between residues 48–200; these data are consistent with the region identified in LBP-9 [13]. A previous characterisation of CRTR-1 as a repressor, based on GAL4 assays [3], mapped the region responsible for repression to residues 1–52 of CRTR-1 and saw no repression mediated by residues 47–479. Unlike that study, we were unable to detect repression with the 1–52 region and saw repression with the C-terminal portion of the protein. Sequencing of the original constructs revealed a number of mutations, although the 1–52 construct appeared to be correct. The consistency of the data presented here with the analysis of LBP-9 and a second study that uses the CRTR-1 1–52 construct and detects only minor repression [2] supports the positioning of the repression domain between residues 48–200.

The E3 sumo ligase PIAS1 has been shown previously to interact with CP2 and affect its transcriptional activity [2]. However, this is the first direct demonstration that CP2 family members can be sumoylated and that this modification appears to affect activity of the protein. Immunoprecipitation experiments showed that CRTR-1 can be sumoylated and that the major site of SUMO-1 conjugation is K30. In addition to the predominant SUMO-1-CRTR-1 bands of approximately 75–80 kD, several higher molecular weight bands were also detected. These may correspond to sumoylation at multiple sites, possibly K464 or other non-consensus sites, as has been observed for Smad4 [16], with conjugation at K30 required for efficient conjugation at subsequent sites. A second possibility is that the higher bands represent polysumoylated forms. Although SUMO-1 lacks a consensus sumoylation sequence, polySUMO-1 chains have been reported to form on RanBP2 [17]. Mutation of residue K30 of CRTR-1 was sufficient to abolish detectable sumoylated CRTR-1 in COS-1 cells and resulted in increased activity in both ES and COS-1 cells, suggesting that sumoylation at K30 blocks maximal transactivation by CRTR-1. This is consistent with a repressive role for sumoylation that is a common consequence of transcription factor sumoylation [18]. The activity of CRTR-1 K30A in COS-1 cells was significantly greater than that of wild type CRTR-1, which had little activity in this cell type, suggesting that this limited activity may be due to higher level sumoylation in COS-1 cells compared to HEK293T or ES cells. Interestingly, despite the higher activity of K30A CRTR-1, the overall level of transcriptional activation observed when K30A was co-expressed with other CP2 family members was generally similar to that seen when other family members were co-expressed with wild type CRTR-1 in both COS-1 and ES cells. One possible explanation for this may be that high level expression of the other CP2 proteins in our assays may saturate machinery required for transcriptional activation of the reporter gene to a maximal level. Alternatively, CRTR-1 sumoylation may interfere with heteromer formation and activity. This effect can be observed when CRTR-1 alone is transfected (compare the activity of CRTR-1 and CRTR-1 K30A; Figure 7A,B), but is undetectable when other family members are co-transfected due to the high level expression of the other family members driving the equilibrium of complex formation to the heteromeric state. The effect on CRTR-1 activity could be mediated by a decrease in DNA binding on sumoylation, as has been reported for Sox2 and Heat Shock Factors [19], [20], by recruitment of co-repressors [18], or by altering subcellular localisation such that CRTR-1 is sequestered and unavailable, such as occurs with Sp3 and ELK-1 [21], [22].

The amino terminal sumoylation sequence of CRTR-1, FKQE, is conserved in mouse CP2, NF2d9 and altNF2d9 proteins, suggesting that these family members may also be sumoylated. However, CRTR-1 has a putative phosphorylation-dependent sumoylation motif (PDSM) [23] adjacent to the K30 residue which is absent in the other proteins (and is also absent from the putative CRTR-1 K464 sumoylation motif). Whether the presence or absence of this motif influences sumoylation of CRTR-1 may provide insight into both the role of sumoylation and its regulation of this family of proteins.

The key findings of this work are that CRTR-1 is able to activate transcription, contrary to previous reports, form DNA-binding heteromers with other CP2 family members and modulate their activity. This implies that these transcription factors should be studied as a family, depending on the complement of family members expressed in a given cell type or tissue of interest. In the case of CRTR-1, gene targeting has identified an important role for this protein in the kidney [4], [5], but it also suggested that expression of CRTR-1 is not critical in the inner cell mass of the blastocyst and ES cells. However, CP2, NF2d9 and altNF2d9 are also expressed in ES cells [2]. The recent findings that CRTR-1 binds to key pluripotency factor regulatory regions [10] suggests that CP2 family complexes should be examined to determine the function of this family in pluripotent cells.

## Materials and Methods

### Plasmid constructs

PCR amplification from mouse kidney cDNA or D3 ES cell cDNA was used to generate fragments encoding the full-length open reading frame of CRTR-1, CP2, NF2d9 and altNF2d9. Note the CRTR-1 sequence corresponds to GenBank Accession number NM_023755. FLAG tags were incorporated on the 5′ end where indicated. XhoI and NotI restriction sites were incorporated on the 5′ and 3′ ends respectively and used to clone the fragments into XhoI/NotI-digested pEF-IRES-puro6 vector [24] to generate pEF-CRTR-1, pEF-FLAG-NF2d9 and pEF-FLAG-altNF2d9. The CP2 expression vector, pEF-FLAG-CP2, was generated via incorporation of an EcoRI site in the forward primer and NotI site in the reverse primer and subsequent cloning into EcoRI/NotI-digested pEF-IRES-puro6 vector. The pEF-FLAG-SUMO-1 plasmid was generated by PCR amplification of the FLAG-SUMO-1 open reading frame from pCMV-FLAG-SUMO-1 [25] with incorporation of XhoI and NotI sites as above for cloning into pEF-IRES-puro6. CRTR-1 sumoylation site mutations K30A and K464A and the double mutation K30A/K464A (2KA) were generated in pEF-CRTR-1 using QuikChange Site-Directed Mutagenesis (Stratagene) and the following primer pairs: K30A 5′-GCT CTG CCT ATC TTC GCA CAG GAA GAG CCG CAG C-3′ and 5′-GCT GCG GCT CTT CCT GTG CGA AGA TAG GCA GAG C-3′; K464A5′-CCT CAG CAC ATT AGC AGC AGA AAG CAA TGA TGG C-3′ and 5′-GCC ATC ATT GCT TTC TGC TGC TAA TGT GCT GAG G-3′. The GAL4-CRTR-1 deletions are in-frame fusions between the yeast GAL4 DNA-binding domain (amino acids 1-147) and fragments of CRTR-1. These constructs were generated in the pGALO plasmid [26] after PCR amplification of the full-length open reading frame or various regions of CRTR-1 as specified. All PCR primers were designed to incorporate SacI and SalI restriction enzyme sites at the 5′ and 3′ ends respectively, permitting cloning into SacI/SalI-digested pGALO vector. The following oligonucleotides were used to generate the constructs specified: pGAL4-CRTR-1 5′-ATA GTC GAC CAG CCA TGC TGT TCT GGC-3′ and 5′-AGA GCT CAC TCA TTC TGC TTA AAC GTG TC-3′; pGAL4-CRTR-1(1–47) 5′-ATA GTC GAC CAG CCA TGC TGT TCT GGC-3′ and 5′-AGA GCT CAT AGG GGC GGC AAG CGG GC-3′; pGAL4-CRTR-1(48-479)5′-CGC GTC GAC AGT ATG TGT TGT GTG CCG C-3′ and 5′-AGA GCT CAG AGT CCA CAC TTC AGG ATG-3′; pGAL4-CRTR-1(1–100) 5′-ATA GTC GAC CAG CCA TGC TGT TCT GGC-3′ and 5′-AGA GCT CAG ATG ATG CTC TTC ACA TAT TTC G-3′; pGAL4-CRTR-1(1-200) 5′-ATA GTC GAC CAG CCA TGC TGT TCT GGC-3′ and 5′-AGA GCT CAC TCA TTC TGC TTA AAC GTG TC-3′; pGAL4-CRTR-1(101–479) 5′-ATC GTC GAC GTG TCG TTT TCC ATG ACC G-3′ and 5′-AGA GCT CAG AGT CCA CAC TTC AGG ATG-3′; pGAL4-CRTR-1(198–479) 5′-CGC GTC GAC AGA ATG AGA GTG GGG ACT AC-3′ and 5′-AGA GCT CAG AGT CCA CAC TTC AGG ATG-3′; pGAL4-CRTR-1(101–200) 5′-ATC GTC GAC GTG TCG TTT TCC ATG ACC G-3′ and 5′-AGA GCT CAC TCA TTC TGC TTA AAC GTG TC-3′; pGAL4-CRTR-1(48–200) 5′-CGC GTC GAC AGT ATG TGT TGT GTG CCG C-3′ and 5′-AGA GCT CAC TCA TTC TGC TTA AAC GTG TC-3′. These constructs were assayed for activity using the GAL4-responsive luciferase reporter construct pTK-MH100x4-LUC [26]. The CP2-responsive luciferase reporter construct pTK-4xWT-CP2-Luc was generated by HindIII/BamHI digestion of pTK-MH100x4-LUC to remove the GAL4-responsive element which was subsequently replaced with 2 sets of double stranded oligonucleotides (5′-ATA GTC GAC CAG CCA TGG CCT GGG CTC TGA AG-3′/5′-ATA AAG CTT GAG CTC CTA CTT GAG AAT GAC ATG-3′ and 5′-ATA GTC GAC CAG CCA TGG ATA GAG AGA AAA TGG AG-3′/5′-ATA AAG CTT GAG CTC ACA AAC TTG ACT CTT CTT G-3′) constituting 4 copies of the α-globin CP2 response element [27]. PIAS1 and Ubc9 full-length open reading frames, originally PCR amplified from mouse kidney cDNA, were FLAG-tagged at the amino terminus and cloned into pEF-IRES.puro6 to generate pEF-FLAG-Ubc9 and pEF-FLAG-PIAS1. pXMT2 [28], pXMT2.PIAS1 and pXMT2.Ubc9 were gifts from Steven Rodda (University of Adelaide).

### Antibodies

CRTR-1-specific rabbit polyclonal antiserum was generated using a 21 amino acid peptide of the CRTR-1 amino-terminus (MLFWHTQPEHYNQHNSGSYLR) conjugated to the carrier diphtheria toxoid (Mimotopes). This sequence is common to CRTR-1 and LBP-9, but is not present in other CP2 family members. Affinity purification of the antibody was performed using the inoculating peptide. Mouse FLAG M2 antibody was obtained from Sigma and used at a dilution of 1∶500. Anti-GAL4(DBD) rabbit polyclonal antibody sc-577 was purchased from Santa Cruz and used at 1∶500. Anti-rat alpha-tubulin (MCA78G) was purchased from Serotec and used at a dilution of 1∶2000. Anti-mouse and anti-rabbit alkaline phosphatase secondary antibodies (used at 1∶2000 dilution) were obtained from Rockland. HRP-conjugated anti-rat secondary antibody (P0450) was purchased from Dako and used at a dilution of 1∶10000.

### Cell culture and luciferase assays

COS-1 and HEK293T cells (CRL-1650 and CRL-11268; ATCC, Manassas, USA) were routinely maintained in DMEM (Invitrogen) supplemented with 10% FCS. D3 ES cells [29] were maintained in DMEM supplemented with 1000 U/ml leukaemia inhibitory factor, 0.1 mM 2-mercaptoethanol and 10% FCS. COS-1, HEK293T and ES cells were seeded at 3.5×10^4^, 5×10^4^ or 3.5×10^4^ cells/well of 24-well trays (Falcon) respectively. COS-1 and HEK293T cells were transfected 16-24 h after seeding and ES cells were transfected 1 h after seeding. Triplicate wells were transfected with 200 ng/well pTK-4xWT-CP2-Luc or pTK-MH100x4-LUC reporter plasmid and 5 ng/well pRL-SV40 renilla plasmid using FuGene 6 (Roche), according to the manufacturer's instructions. The total amount of DNA transfected was standardised to 605 ng with the appropriate empty vector. Cell extracts were assayed 40 h post-transfection using the Dual-Luciferase reporter assay system (Promega) according to the manufacturer's instructions. Relative luciferase activity was determined as a ratio of Firefly/Renilla luciferase levels and data were expressed as the mean (± SEM) of triplicate values obtained from a representative experiment that was independently repeated at least 3 times, unless otherwise stated. When data from multiple experiments were combined, statistical analysis was performed using the two-tailed unpaired t-test. A value of P<0.05 was considered statistically significant.

### Coimmunoprecipitation, sumoylation and western blot analyses

For co-immunoprecipitation studies, HEK293T or COS-1 cells were plated at a density of 1×10^6^ or 2.5×10^5^ cells per 6 cm dish respectively. Cells were transfected with equal amounts (1 µg) of plasmid encoding CRTR-1 and CP2 family members. The total amount of DNA transfected was made up to 2 µg with pEF-IRES-puro6. Cells were lysed 40 h post-transfection in 1 ml IP lysis buffer [50 mM Tris-HCl pH 7.5, 150 mM NaCl, 10% (v/v) glycerol, 1% Triton X-100, 10 mM EDTA, 1 X protease inhibitor cocktail (Roche)]. Lysates were incubated with 10 µg anti-CRTR-1 affinity purified antibody or 20 µg anti-FLAG M2 antibody (Sigma) for 3 h at 4°C and immunoprecipitated by incubation with 50 µl protein-A agarose beads (Roche). Immunoprecipitated proteins were eluted from the beads by boiling in 25 µl 2X SDS load buffer [125 mM Tris-HCl pH 6.8, 4% (v/v) SDS, 20% (v/v) glycerol, 0.1% (w/v) bromophenol blue, 5% (v/v) 2-mercaptoethanol] for 5 min, resolved by SDS-PAGE and subjected to western blot analysis. For detection of sumoylated proteins, COS-1 cells were plated at 5×10^5^ cells per 10 cm dish and transfected with plasmids encoding CRTR-1 (3 µg) and FLAG-SUMO-1 (3 µg). The total amount of DNA transfected was made up to 6 µg with pEF-IRES-puro6. Cells were lysed in 1 ml IP lysis buffer [50 mM Tris-HCl pH 7.5, 150 mM NaCl, 10% (v/v) glycerol, 1% Triton X-100, 10 mM EDTA) containing 1x protease inhibitor cocktail (Roche), 10 mM Iodoacetamide (Sigma), and 10 mM N-ethylmaleimide (NEM; Sigma)]. FLAG-SUMO-1 conjugated proteins were immunoprecipitated using 20 µl anti-FLAG M2 affinity gel according to the manufacturer's protocol (Sigma). CRTR-1 proteins were immunoprecipitated as described above and analysed by western blotting.

### Electrophoretic mobility shift assays

HEK293T cells (1.5×10^6^ cells/10 cm dish) and COS-1 cells (5×10^5^/10 cm dish) were transfected with plasmids encoding CRTR-1 (3 µg) and CP2 family members (3 µg). Total DNA transfected was made up to 6 µg with pEF-IRES-puro6. Cells were lysed 40 h post-transfection with 2.5 pellet volumes of lysis buffer A [10 mM HEPES, pH 7.9, 1.5 mM MgCl_2_, 10 mM KCl, 0.4% Igepal (Sigma), 10% Ficoll-400, 1 mM PMSF, 1x protease inhibitor cocktail (Roche), 1 mM DTT]. Following removal of the cytosolic fraction, the nuclear fraction was lysed in 1.5 pellet volumes of lysis buffer B [20 mM HEPES, pH 7.9, 1.5 mM MgCl_2_, 0.5 mM EDTA, 20% glycerol, 0.42 M KCl, 1 mM PMSF, 1 mM DTT, 1x protease inhibitor cocktail]. Protein-DNA binding reactions contained 7–10 µg nuclear protein extract, 1x gel-shift binding buffer [10 mM Tris-HCl pH 8.0, 10% glycerol, 2% PVA, 0.1 mM EDTA, 100 mM KCl and 1 mM DTT], 1 µg Poly(dI-dC) and 20 nM carboxyfluorescein (FAM)-labelled annealed oligonucleotides in a total volume of 20 µl. Reactions were incubated at room temperature for 30 min prior to electrophoresis on a 0.5x TBE buffered 4% PAGE gel that had been pre-electrophoresed for 2 h. Electrophoresis was performed at at a constant voltage of 200V for 2.5 h and results were detected using a Typhoon Trio Variable Mode Imager (Amersham Biosciences). For supershift analysis, 4 µg anti-CRTR-1 affinity purified antibody or pre-immune serum was incubated with 7-10 µg nuclear protein extract for 15 min on ice prior to addition of the DNA probe. Sequence of the double-stranded wild type mouse α-globin CP2-binding site oligonucleotide is 5′-TCG AGC AAG CAC AAA CCA GCC AAC-3′ and that of the mutant CP2-binding site is 5′-TCG AGA AAT CAC AAA ACA TCC AAC-3′ [27].

## Supporting Information

Figure S1 A. CRTR-1 activates transcription in ES cells. Cells were co-transfected with pTK-4xWT-CP2-LUC and pEF-CRTR-1 expression plasmid (CRTR-1) as indicated. Relative luciferase activity was determined by normalisation to renilla activity. The data are presented relative to the activity of the reporter vector alone and are the mean ±SEM of two independent experiments, each of which was conducted in triplicate. Note: fold activation seen here is higher than that seen in the comparable experiments in Figure 1. Our evidence suggests that this difference is likely to be due to a change in DNA preparation method between the experiments. Endotoxin-free DNA was used for all transfections, apart from those in S1A. B. Western blot analysis of ES, HEK293T and COS-1 cells transfected with pTK-4xWT-CP2-LUC and pEF-CRTR-1 expression plasmid (CRTR-1) as indicated. Cells were lysed 48 h post-transfection and 20% of total cell lysate was analysed. The upper panel shows membrane probed with rabbit anti-LBP-9 antibody (LS-C30155, LifeSpan Biosciences, 1:500) and detected using enhanced chemifluorescence. The lower panel is a re-probing of the membrane with rat anti-alpha-tubulin antibody and detection using chemiluminescence.
(0.38 MB TIF)

Figure S2 Western blot analysis of CRTR-1, FLAG-CP2, FLAG-NF2d9 and FLAG-altNF2d9 expression levels. Western blot analyses were performed on 30% of the HEK293T cell lysate from experiments included in Fig. 5B. Cells were transfected with the appropriate expression plasmids, as indicated (ng). Proteins were detected using ECF and anti-CRTR-1 or anti-FLAG (M2) antibodies.
(1.41 MB TIF)

Figure S3 A. This is a re-probing of the blot from Fig. 6C with anti-CRTR-1 antibody to demonstrate that CRTR-1 is successfully immunoprecipitated and that levels of wild-type and sumoylation-mutant CRTR-1 proteins are expressed at comparable levels. B. Western blot analysis of ES cells transfected with the indicated amounts of pEF-CRTR1, pEF-K30A, pEF-K2A or pEF-K464A and pTK-4xWT-CP2-LUC reporter construct (ng). Cells were lysed 48 h post-transfection and 20% of total cell lysate was analysed. The upper panel shows membrane probed with rabbit anti-LBP-9 antibody (LS-C30155, LifeSpan Biosciences, 1∶500) to detect CRTR-1 (arrow) using enhanced chemifluorescence. The lower panel is re-probing of the membrane with rat anti-alpha-tubulin antibody and detection using chemiluminescence.
(0.33 MB TIF)
