# Supplementary figures and images for: Modulation of CP2 Family Transcriptional Activity by CRTR-1 and Sumoylation
Source: PLoS One. 2010 Jul 22;5(7):e11702. doi: 10.1371/journal.pone.0011702 (PMC2908540; doi:10.1371/journal.pone.0011702)

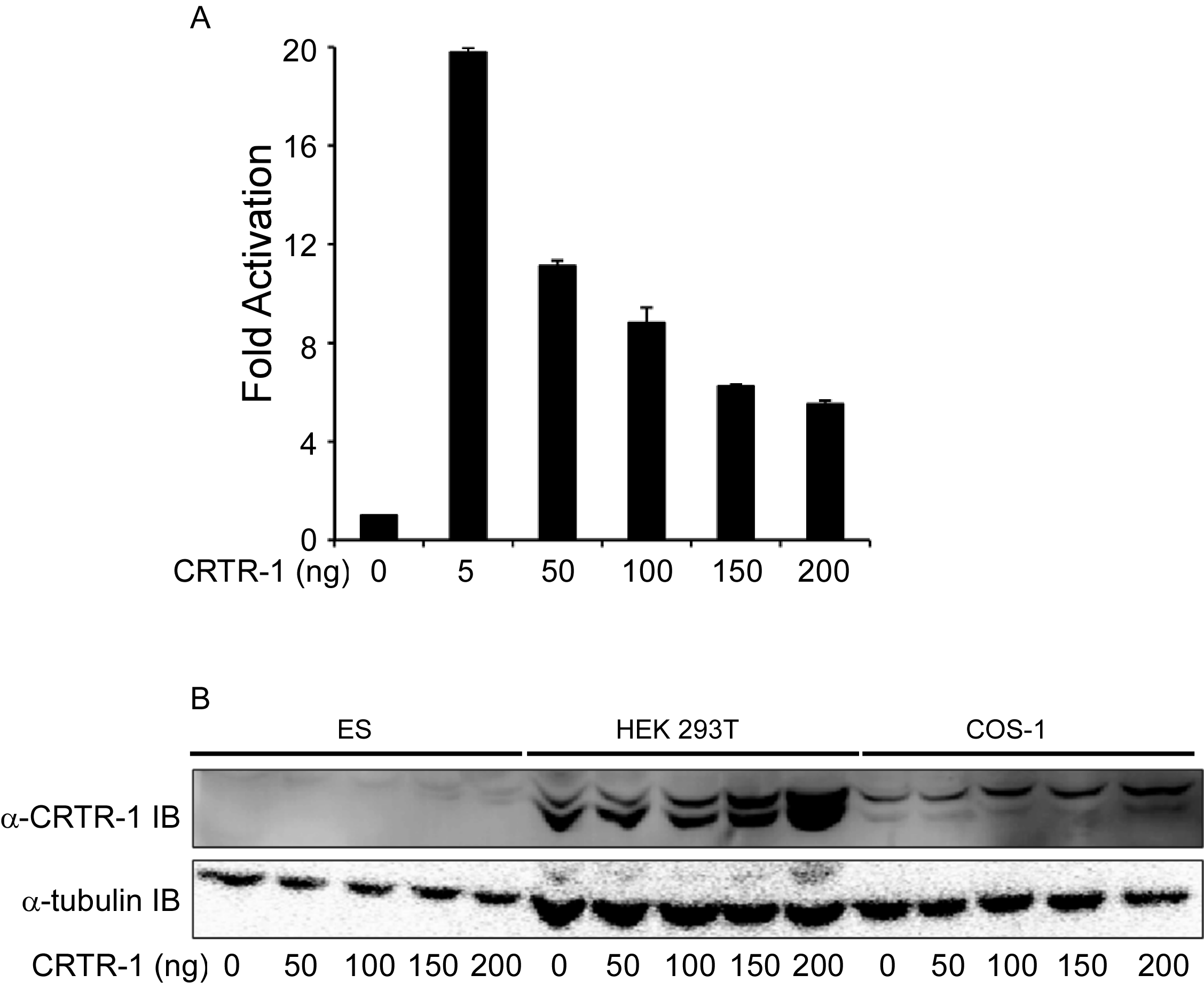

Supplement: Figure S1 — A. CRTR-1 activates transcription in ES cells. Cells were co-transfected with pTK-4xWT-CP2-LUC and pEF-CRTR-1 expression plasmid (CRTR-1) as indicated. Relative luciferase activity was determined by normalisation to renilla activity. The data are presented relative to the activity of the reporter vector alone and are the mean ±SEM of two independent experiments, each of which was conducted in triplicate. Note: fold activation seen here is higher than that seen in the comparable experiments in Figure 1. Our evidence suggests that this difference is likely to be due to a change in DNA preparation method between the experiments. Endotoxin-free DNA was used for all transfections, apart from those in S1A. B. Western blot analysis of ES, HEK293T and COS-1 cells transfected with pTK-4xWT-CP2-LUC and pEF-CRTR-1 expression plasmid (CRTR-1) as indicated. Cells were lysed 48 h post-transfection and 20% of total cell lysate was analysed. The upper panel shows membrane probed with rabbit anti-LBP-9 antibody (LS-C30155, LifeSpan Biosciences, 1:500) and detected using enhanced chemifluorescence. The lower panel is a re-probing of the membrane with rat anti-alpha-tubulin antibody and detection using chemiluminescence. (0.38 MB TIF) [file pone.0011702.s001.tif]

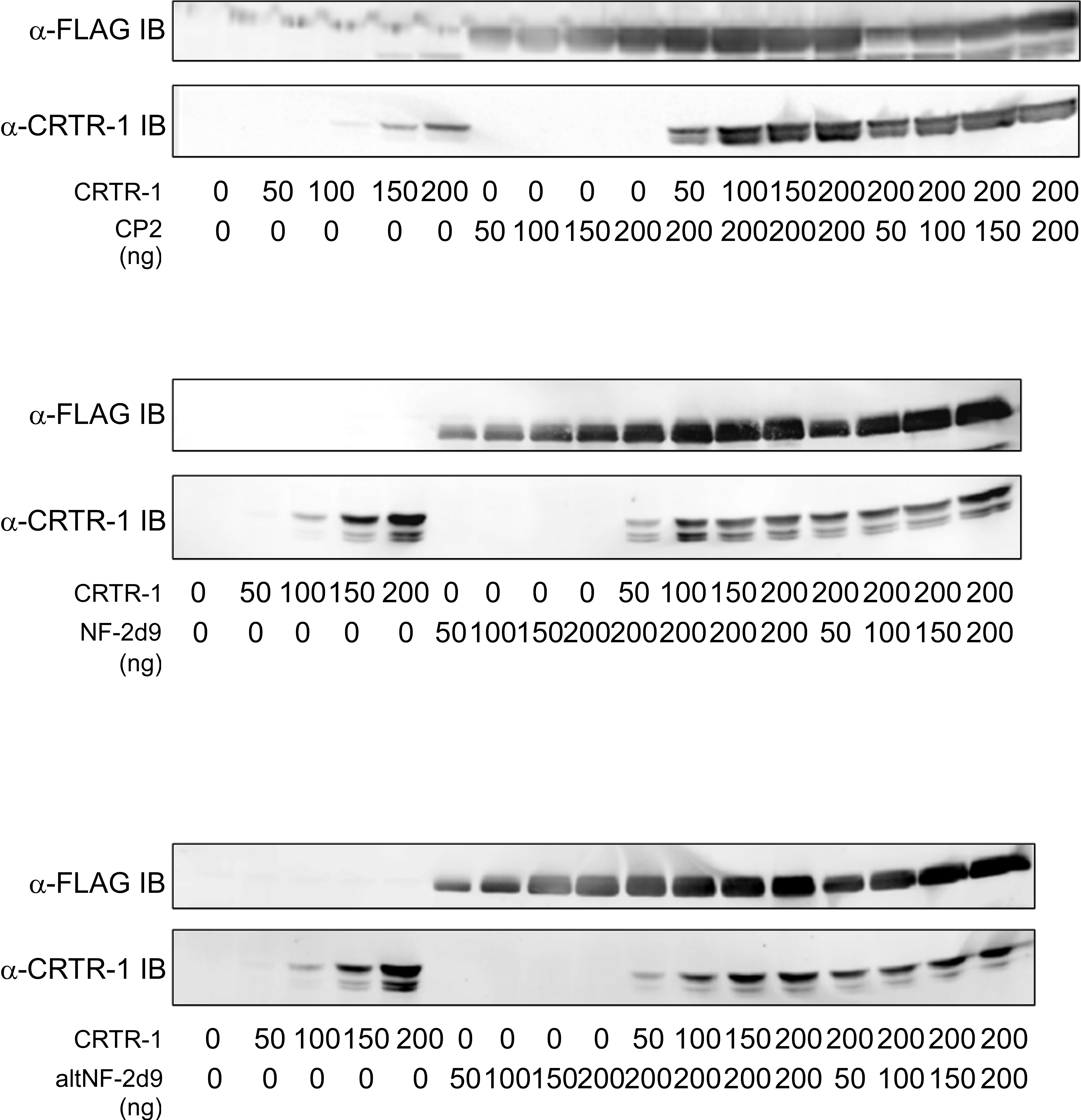

Supplement: Figure S2 — Western blot analysis of CRTR-1, FLAG-CP2, FLAG-NF2d9 and FLAG-altNF2d9 expression levels. Western blot analyses were performed on 30% of the HEK293T cell lysate from experiments included in Fig. 5B. Cells were transfected with the appropriate expression plasmids, as indicated (ng). Proteins were detected using ECF and anti-CRTR-1 or anti-FLAG (M2) antibodies. (1.41 MB TIF) [file pone.0011702.s002.tif]

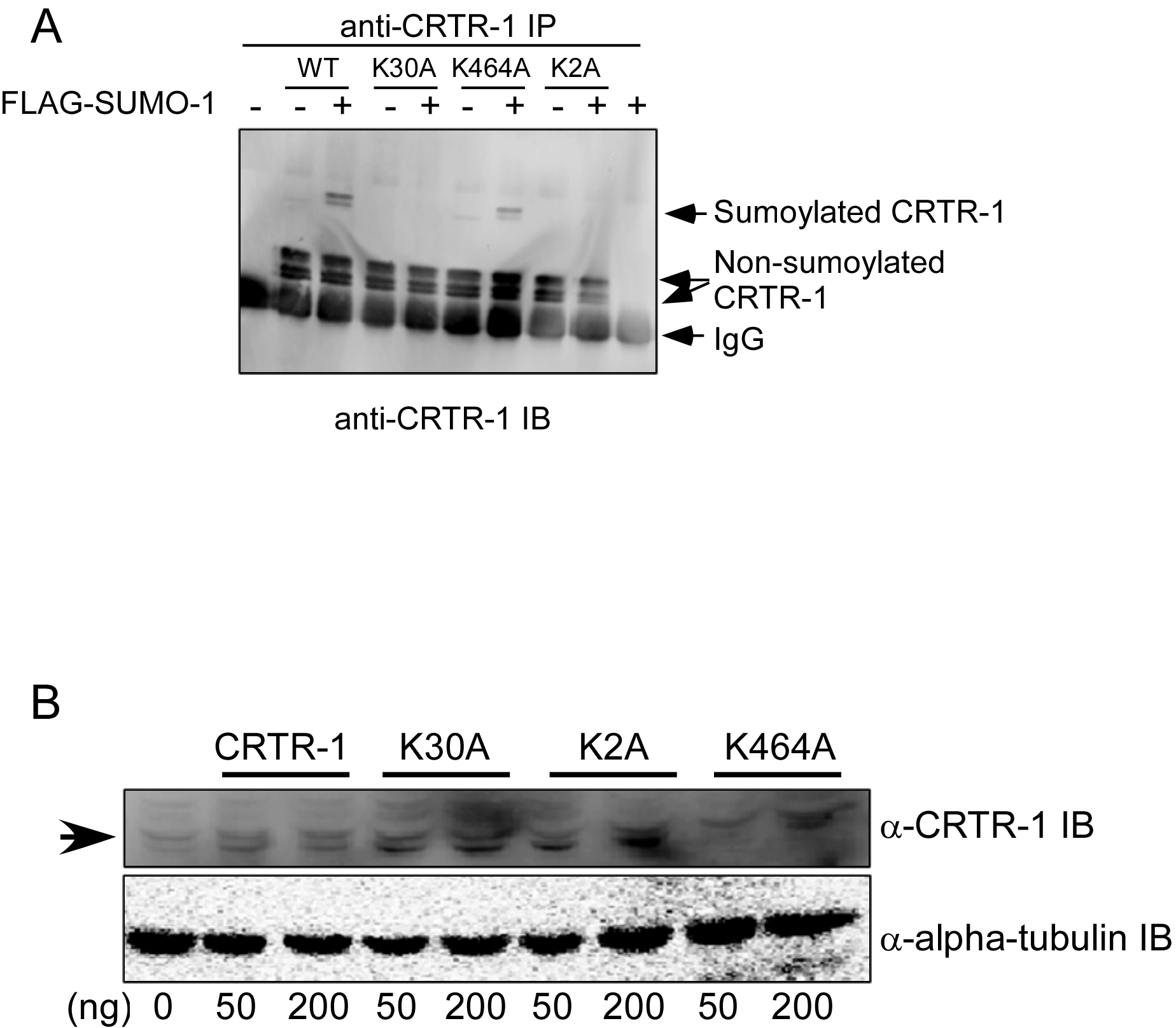

Supplement: Figure S3 — A. This is a re-probing of the blot from Fig. 6C with anti-CRTR-1 antibody to demonstrate that CRTR-1 is successfully immunoprecipitated and that levels of wild-type and sumoylation-mutant CRTR-1 proteins are expressed at comparable levels. B. Western blot analysis of ES cells transfected with the indicated amounts of pEF-CRTR1, pEF-K30A, pEF-K2A or pEF-K464A and pTK-4xWT-CP2-LUC reporter construct (ng). Cells were lysed 48 h post-transfection and 20% of total cell lysate was analysed. The upper panel shows membrane probed with rabbit anti-LBP-9 antibody (LS-C30155, LifeSpan Biosciences, 1∶500) to detect CRTR-1 (arrow) using enhanced chemifluorescence. The lower panel is re-probing of the membrane with rat anti-alpha-tubulin antibody and detection using chemiluminescence. (0.33 MB TIF) [file pone.0011702.s003.tif]
